# Return to Work for Mental Ill-Health: A Scoping Review Exploring the Impact and Role of Return-to-Work Coordinators

**DOI:** 10.1007/s10926-020-09873-3

**Published:** 2020-01-30

**Authors:** E. MacEachen, E. McDonald, E. Neiterman, E. McKnight, C. Malachowski, M. Crouch, S. Varatharajan, N. Dali, E. Giau

**Affiliations:** School of Public Health and Health Systems, University of Waterloo, 200 University Avenue West, Waterloo, ON N2L 3G1 Canada

**Keywords:** Mental health, Return to work, Return to work coordinators, Scoping review, Case management

## Abstract

*Purpose* This scoping review was completed to explore the role and impact of having a return-to-work (RTW) coordinator when dealing with individuals with common mental ill-health conditions. *Methods* Peer reviewed articles published in English between 2000 and 2018 were considered. Our research team reviewed all articles to determine if an analytic focus on RTW coordinator and mental ill-health was present; consensus on inclusion was reached for all articles. Data were extracted for all relevant articles and synthesized for outcomes of interest. *Results* Our search of six databases yielded 1798 unique articles; 5 articles were found to be relevant. The searched yielded only quantitative studies. Of those, we found that studies grouped mental ill-health conditions together, did not consider quality of life, and used different titles to describe RTW coordinators. Included articles described roles of RTW coordinators but did not include information on their strategies and actions. Included articles suggest that RTW interventions for mental ill-health that utilize a RTW coordinator may result in delayed time to RTW. *Conclusions* Our limited findings suggest that interventions for mental ill-health that employ RTW coordinators may be more time consuming than conventional approaches and may not increase RTW rate or worker’s self-efficacy for RTW. Research on this topic with long-term outcomes and varied research designs (including qualitative) is needed, as well as studies that clearly define RTW coordinator roles and strategies, delineate results by mental health condition, and address the impact of RTW coordinators on workers’ quality of life.

## Introduction

Health-related absence from the workplace concerns many stakeholders and in different ways: employers are concerned with loss of productivity, workers with health and financial security, and social welfare systems with cost. Internationally, mental ill-health (including both mental illness and mental health concerns such as distress and burnout) impacts one in five adults each year and is one of the leading causes of disability and absence from the workplace [1, 2]. In Canada, mental ill-health accounts for 30% of short and long-term disability claims in Canada [3] and, in the US, is associated with increased work absence and unemployment [4]. Estimates suggest the financial burden associated with lost productivity, increased presenteeism and workplace absences in Canada is $6 billion annually [1, 3] and over $2 billion in the US for depression alone [4].

Return-to-work (RTW) coordinators—individuals employed to plan and facilitate the return to work of workers who are absent from work due to injury or illness—are increasingly seen as an important resource for helping workers to navigate RTW trajectories, which can be complex. Although there is no clear definition for their role, these individuals engage with various parties and processes involved with RTW, including health care, supervisors and insurance companies, in order to guide workers back to work [5]. RTW coordinators can occupy different positions within or outside of the workplace hierarchy. For instance, they may be housed in the human resources arm of a large company, be independent consultants to businesses, or be case managers in insurance and workers’ compensation organisations [6]. As all RTW stakeholders (i.e. employers, workers, health care providers) operate from distinct economic, social and legislative contexts, RTW coordinators may facilitate communication between parties that, at times, have conflicting goals and objectives [7, 8].

RTW coordination is especially complex when dealing with mental health cases. A symptom of mental ill-health is decreased motivation to engage in society, but motivation is considered to be key to RTW [7]. Further, societal stigma surrounding people with mental ill-health may pose a barrier for re-entry to the workplace. Thus, RTW coordinators face particular challenges when dealing with workplace absences due to mental ill-health.

While there is a developing body of literature about RTW coordination and its value, there are limited reviews focusing on the roles, strategies and actions completed by RTW coordinators [9]. This is especially true when considering RTW coordination for individuals with mental ill-health, where unique strategies may be required to meet the needs of individuals and overcome potential barriers associated with some mental ill-health conditions. As a result, this article reports on a scoping review of RTW coordinators and mental health. In particular, we focused on the following questions:

1. How is the role of RTW coordinators and mental health conceptualised in academic literature?

2. What strategies and actions do RTW coordinators use to manage mental health cases?

3. What is the impact of RTW coordinators on the RTW outcomes and experiences of individuals with mental ill-health?

## Methods

We completed a scoping review to address our research questions because it allowed for consideration of the complexity of the RTW arena, including the variety of players and definitions present and did not restrict the focus to a particular method.

This review is derived from a larger review [10] being completed by our research team that aims to assess all peer-reviewed literature, published between January 1, 2000 and March 23, 2018 that focused on the strategies, actions and/or impact of RTW coordinators managing work reintegration for any health concern. The research team included individuals with a strong understanding of RTW coordination, mental health, and with previous experience conducting systematic reviews.

A systematic approach was taken to search, critically assess the literature and distill the findings. First, research questions were clarified among the research team and a comprehensive search strategy was created. Once articles had been retrieved, inclusion and exclusion criteria were again clarified, and articles were screened for their relevance to our topic.

The following working definitions were used to guide article screening:

### RTW Coordinators

Articles were required to have an analytic focus not simply on RTW coordination but on the strategies, actions and/or impact of RTW coordinators. Coordinators were defined as individuals whose job involved facilitating appropriate and timely RTW of workers who had a workplace absence due to illness or injury.

### Mental Ill-Health

Relevance for mental ill-health was limited to studies that included RTW coordination for participants facing common mental health challenges. These included clinical concerns such as anxiety and depression, as well as sub-clinical concerns, such as burnout and distress. As the RTW trajectory for those with serious mental health concerns, such as bi-polar disorder, schizophrenia or psychosis, is unique, studies focusing on or including individuals with these health conditions were excluded [11].

### Inclusion and Exclusion Criteria

Based on the language proficiency of our research team, articles not published in English were excluded. Research designs could be qualitative or quantitative, mixed methods, or scoping and systematic review articles. Articles that did not contain any empirical findings, such as editorials, opinion pieces and literature reviews, were excluded. Articles had to have research outcomes for both mental health and RTW coordinators. Articles where the strategies, actions and/or impact of the RTW coordinator could not be disentangled from other aspects of the intervention were not included.

### Search Terms

Search terms for our review were selected by examining keywords from similar published articles and by consulting with a university librarian. Six databases were searched for articles published between January 1, 2000 and March 23rd, 2018. These databases were: American Business Index (ABI) Inform, CINAHL, Embase, MEDLINE, PsychINFO and Web of Science (see Table 1 for search terms).

**Table 1:** Literature search strategies

Database	Search strategy
MEDLINE	1. Return to work/ 2. Rehabilitation, vocational/ 3.Return* to work*.ab,ti. 4. RTW.ab,ti. 5. Work accommodation*.ab,ti. 6. Job accommodation*.ab,ti. 7. Disability management.ab,ti. 8. Absence management.ab,ti. 9. Or/1–8 10. Coordinator*.ab,ti. 11. Co ordinator*.ab,ti. 12. Coordination*.ab,ti. 13. Co ordination*.ab,ti. 14. Case manager*.ab,ti. 15. Disability manager*.ab,ti. 16. Case management/ 17. Or/10–16 18. 9 and 17 19. limit 18 to (english language and year = "2000-Current")
EMBASE	1. Return to work/ 2. Vocational rehabilitation/ 3. Return* to work*.ab,ti. 4. RTW.ab,ti. 5. Work accommodation*.ab,ti. 6. Job accommodation*.ab,ti. 7. Disability management.ab,ti. 8. Absence management.ab,ti. 9. Or/1–8 10. Coordinator*.ab,ti. 11. Co ordinator*.ab,ti. 12. Coordination*.ab,ti. 13. Co ordination*.ab,ti. 14. Case manager*.ab,ti. 15. Disability manager*.ab,ti. 16. Case management/ 17. Or/10–16 18. 9 and 17 19. limit 18 to (english language and year = "2000 -Current") 20. 19 NOT conference abstract.pt
CINAHL	S1 MH Job re-entry S2 MH “Rehabilitation, vocational + ” S3 Return* to work* S4 RTW S5 Work accommodation* S6 Job accommodation* S7 Disability management S8 Absence management S9 Or/S1-S8 S10 Coordinator* S11 Co ordinator* S12 Coordination* S13 Co ordination* S14 Case manager* S15 Disability manager* S16 MH Case management S17 Or/S10-S16 S18 S9 and S17 (Limiters—English language and year = "January 2000 -March 2018")
PsychINFO	1. Index Terms: {reemployment} OR {Supported Employment} OR{Vocational Evaluation} OR {Work Adjustment Training}rtw OR 2. Any Field: Return* to work* 3. Any Field: RTW 4. Any Field: Work accommodation* 5. Any Field: Job accommodation* 6. Any Field: Disability management 7. Any Field: Absence management 8. Or/1–7 9. Any Field: Coordinator* 10. Any Field: Co ordinator* 11. Any Field: Coordination* 12. Any Field: Co ordination* 13. Any Field: Case manager* 14. Any Field: Disability manager* 15. Index Terms: Case management 16. Or/9–15 17. 8 and 16 and Year: 2000 to 2018
Web of science	1. Topic: (“Return* to work*”) 2. Topic: (RTW) 3. Topic: (Re$employment) 4. Topic: (“Job re$entry”) 5. Topic: (“Work accommodation*”) 6. Topic: (“Job accommodation*”) 7. Topic: (“Disability management”) 8. Topic: (“Absence management”) 9. Combine sets: Or/1–8 10. Topic: (Co$ordinator*) 11. Topic: (Co$ordination*) 12. Topic: (“Case manager*”) 13. Topic: (“Disability manager*”) 14. Topic: (“Case management”) 15. Combine sets: Or/10–14 16. Combine sets: 9 and 15 and language: English (Timespan = 2000–2018)
ABI inform	S1 MAINSUBJECT.EXACT("Return to work programs") S2 MAINSUBJECT.EXACT("Vocational rehabilitation") S3 MAINSUBJECT.EXACT("Disability management") S4 AB,TI(“Return* to work*”) S5 AB,TI(“RTW”) S6 AB,TI(“Work accommodation*”) S7 AB,TI(“Job accommodation*”) S8 AB,TI(“Disability management*”) S9 AB,TI(“Absence management”) S10 MAINSUBJECT.EXACT("Return to work programs") OR MAINSUBJECT.EXACT("Vocational rehabilitation") OR MAINSUBJECT.EXACT("Disability management") OR AB,TI("Return* to work*") OR AB,TI("RTW") OR AB,TI("Work accommodation*") OR AB,TI("Job accommodation*") OR AB,TI("Disability management*") OR AB,TI("Absence management") S11 AB,TI(“Coordinator*”) S12 AB,TI(“Co ordinator*”) S13 AB,TI(“Coordination*”) S14 AB,TI(“Co ordination*”) S15 AB,TI(“Case manager*”) S16 AB,TI(“Disability manager*”) S17 MAINSUBJECT.EXACT("Case management") S18 AB,TI("Coordinator*") OR AB,TI("Co ordinator*") OR AB,TI("Coordination*") OR AB,TI("Co ordination*") OR AB,TI("Case manager*") OR AB,TI("Disability manager*") OR MAINSUBJECT.EXACT("Case management") S19 (MAINSUBJECT.EXACT("Return to work programs") OR MAINSUBJECT.EXACT("Vocational rehabilitation") OR MAINSUBJECT.EXACT("Disability management") OR AB,TI("Return* to work*") OR AB,TI("RTW") OR AB,TI("Work accommodation*") OR AB,TI("Job accommodation*") OR AB,TI("Disability management*") OR AB,TI("Absence management")) AND (AB,TI("Coordinator*") OR AB,TI("Co ordinator*") OR AB,TI("Coordination*") OR AB,TI("Co ordination*") OR AB,TI("Case manager*") OR AB,TI("Disability manager*") OR MAINSUBJECT.EXACT("Case management")) Additional Limits—Date: From 2000 to 2018; Language: English

### Sample Screening

Each article was assigned to varied groups of two research team members who independently evaluated the title and abstract of each article for relevance to RTW coordinators. For articles of uncertain relevance, full manuscripts were reviewed. Research team members met in pairs to review their independent assignments for article relevance. When discrepancies occurred, they were reviewed until consensus was reached. In the event consensus could not be easily reached, the article was flagged and discussed with the entire research team.

### Data Extraction

Data extracted from each article focused on the study purpose, design, findings of interest, strengths and limitations (see Table 2).

**Table 2:** Data extraction components

Data extraction component	Information collected
Study characteristics	Country of intervention; Publication date; MH population included; RTW Coordinator title; Length of observation; Intervention completed
Study purpose	Purpose and/or aim(s) of the study
Methodology	Study design; Sample size; Data analysis approach
Key findings	Strengths and limitations; Primary and secondary outcomes of interest; key findings and conclusions
Findings of interest	Role of RTW coordinators; Strategies and actions completed by RTW coordinators in relation to workers with mental ill-health; Observed MH effect
Critical assessment	Fit with other studies reviewed; Unique insight provided; Aspects overlooked

Two research team members independently extracted data from all relevant articles and then met to compile their individual findings into one consensus document. All consensus data extraction documents were discussed with all study members at research meetings to ensure that information being extracted from each study was relevant and meaningful to the research question.

### Synthesis

Synthesis involved comparing and contrasting studies for conceptualisations of mental ill-health, RTW Coordinators and RTW outcomes, and for strategies and impact of RTW coordinators. We critically considered trends and methodological gaps in the literature.

## Results

### Literature Search and Inclusion

**Fig. 1:**
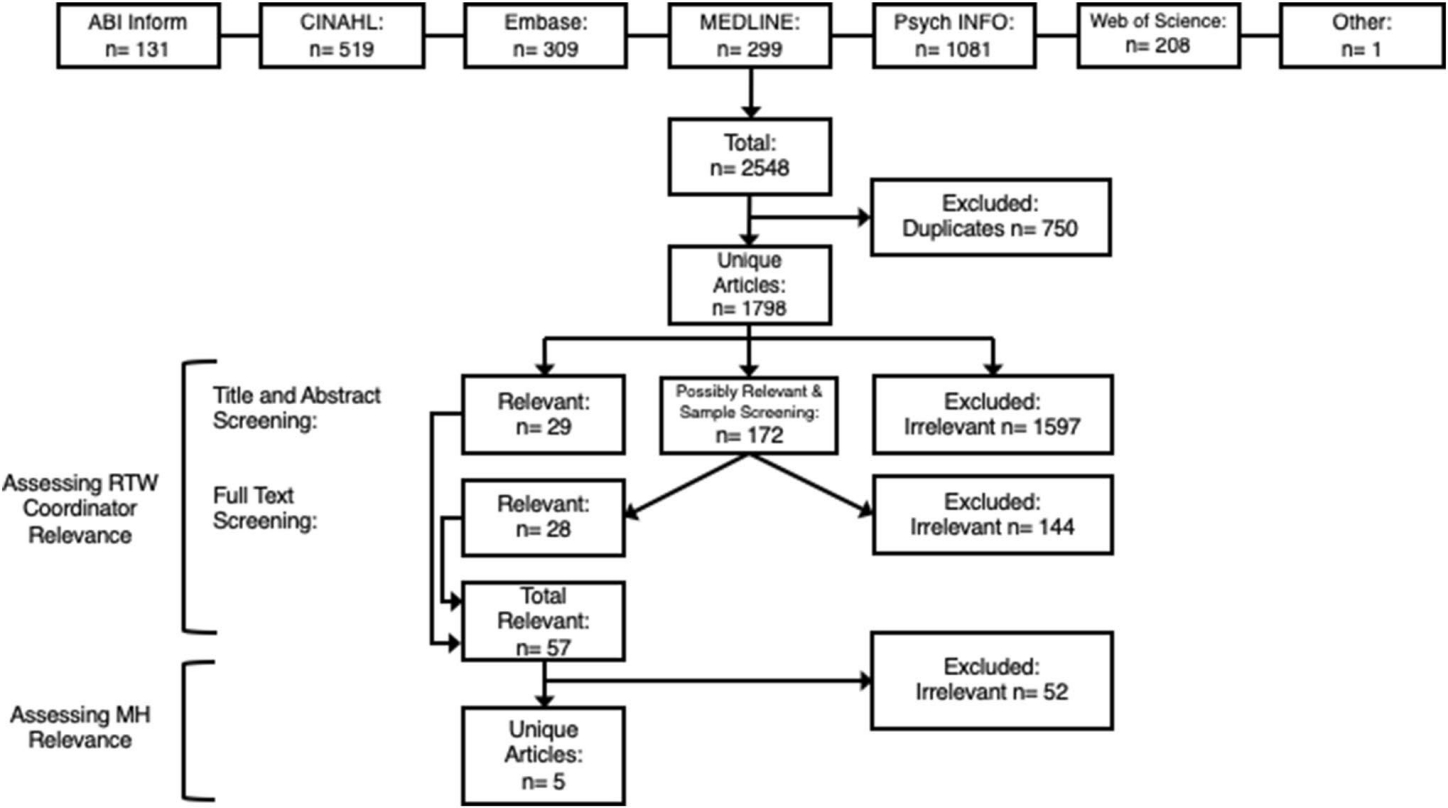
Flowchart of scoping review screening

After merging the results of the literature search from all databases and removing duplicate articles, a total of 1798 unique articles were identified (Fig. 1). These articles were then assessed for relevance based on inclusion criteria. Ultimately, only 5 articles (from 4 studies) met the inclusion criteria for relevance to both RTW coordinators and mental ill-health. These 5 studies (i.e. 4 primary studies and one follow-up study) proceeded to data extraction. It is worth noting that we excluded a significant number of studies that addressed severe mental health conditions, such as bi-polar disorder and schizophrenia. Severe mental health conditions are differentiated from common mental health conditions by their diagnosis, length of duration, and the type of disability they produce, which makes their treatment and prognosis different from that of common mental health conditions [12]. Thus, our focus was on RTW Coordination for less researched, but far more prevalent conditions: common mental health conditions.

A detailed description of the characteristics for all included articles is provided in Table 3. Additionally, the intervention for each study is summarized (Table 4) as are the outcomes of interest from each article (Table 5).

**Table 3:** Description of study characteristics

Author	Year	Location	Study design	Total sample size	Coordinator title	Mental ill-health population included
Black et al. [15]	2017	Australia	Cross-sectional	357	RTW Coordinator	Common mental disorders including stress, depression, anxiety, bullying, and posttraumatic stress disorder
van Oostrom et al. [17]	2009	The Netherlands	Feasibility study of an RCT	40	RTW Coordinator	Individuals with distress, including criteria-based psychiatric disorders (mostly depressive and anxiety disorders) and ‘subthreshold’ disorders (including adjustment disorders)
Lander et al. [16]	2009	Denmark	Controlled interventional study	161	Social Worker	Individuals with emotional distress or common mental health problems
Martin et al. [13]	2013	Denmark	Quasi-randomised control trial	168	Social Worker	Common mental health problems, defined as mood disorders, neurotic, stress-related or somatoform disorders or related conditions e.g. burnout (no co-morbid psychotic conditions)
Martin et al. [14]	2015	Denmark	Quasi-randomised control trial	167	Social Worker	Common mental health problems, defined as mood disorders, neurotic, stress-related or somatoform disorders or related conditions e.g. burnout (no co-morbid psychotic conditions)

**Table 4:** Description of study interventions

Author	Year	Study purpose	Intervention	Length of observation
Black et al. [15]	2017	To identify overall shared and injury specific modifiable factors (including types of contact with RTW Coordinators) associated with self-efficacy in RTW for individuals with upper-body musculoskeletal or psychological injury	45-min computer assisted telephone interview	NA
van Oostrom et al. [17]	2009	To describe the reach and extent of the workplace intervention, the satisfaction and expectation of all stakeholders, and the intention to use the workplace intervention in the future	A stepwise process to identify and solve obstacles for RTW based on consensus between employees and employers facilitated by a RTW coordinator	Start of intervention, 3 months follow-up and completion of workplace intervention
Lander et al. [16]	2009	To evaluate the effect of an intervention program compared to usual welfare benefit care on RTW	Individual consultations with a psychologist and a RTW coordinator	68 weeks
Martin et al. [13]	2013	To assess the effectiveness of a multidisciplinary, coordinated and tailored approach as implemented among individuals with mental health problems	A coordinated team providing a work disability screening, creation and implementation of a RTW plan facilitated by a RTW coordinator for 12 weeks	52 weeks
Martin et al. [14]	2015	To assess the effects of a multidisciplinary, coordinated and tailored RTW-intervention in terms of stability of RTW, cumulative sickness absence and labour market status after 2 years among individuals with mental health problems	A coordinated team providing a work disability screening, creation and implementation of a RTW plan facilitated by a RTW coordinator for 12 weeks	2 years

**Table 5:** Outcomes of interest

Author	Year	Primary outcome	Secondary outcomes	Observed MH effect
Black et al. [15]	2017	RTW self-efficacy (9-item scale) for individuals with physical and mental health concerns	NA	No differences in predictors of RTW self-efficacy between physical and mental injury types Decreased RTW self-efficacy in workers with psychological injury as compared to those with physical injury Increased RTW self-efficacy with low-stress (versus high-stress) contact with a RTW coordinator
van Oostrom et al. [17]	2009	Reach, implementation, satisfaction, expectations, and maintenance regarding the workplace intervention for individuals with mental ill-health	NA	Increased time investment when using a RTW coordinator (compared to the conventional approach) was the only barrier for implementation identified Satisfaction of worker and employer stakeholders was achieved
Lander et al. [16]	2009	Time to RTW for individuals with mental ill-health	NA	No difference in RTW rate between control and intervention groups (with and without RTW coordinators)
Martin et al. [13]	2013	Time to RTW for individuals with mental ill-health	Labor market status	Increased time to RTW in the intervention group (with RTW coordinators) Decreased RTW rate in the intervention group (with RTW coordinators)
Martin et al. [14]	2015	Number of days spent on sickness absence for individuals with mental ill-health	Labor market status	Increased cumulative sickness absence among those in the intervention group (with RTW coordinators) at 1-year and 2-years from RTW Decreased RTW in control group (with no RTW coordinators) at the end of 2-years No difference in recurrent sickness absence or unemployment between groups

Study design and jurisdiction—All of the studies in the final sample used quantitative designs. Two of the articles (both from the same Danish study) gave results for a quasi-randomized control trial [13, 14]. The remaining three articles included: a cross sectional study (Australia) [15], a controlled interventional study (Denmark) [16] and a feasibility study (Netherlands) [17].

Sample size and characteristics—The smallest sample size included 40 participants (feasibility study) [17] and the largest included 357 participants (cross sectional study) [15]. All studies included both males and females of working age.

Inclusion and exclusion criteria—Four of the five studies [13, 14, 16, 17] specifically excluded individuals with concurrent psychotic disorders. The fifth study [15] focused on common mental health disorders and did not specify excluded concurrent conditions. One study also excluded individuals with alcohol or drug abuse concerns [16]. While all studies focused on a population with common mental health concerns, two studies broadened the definition beyond diagnosed mental health concerns and included individuals experiencing emotional distress [16, 17].

RTW Coordinator Title—Two different terms were used describe the RTW coordinator position; three articles referred to these individuals as Social Workers [13, 14, 16] and two articles called these individuals RTW coordinators [15, 17].

RTW Coordinator Role—In two of the articles, RTW coordinators were responsible for assessing employee functioning while exploring the barriers and resources present for RTW in order to form a multidisciplinary RTW plan [13, 14]. Another study identified that the RTW coordinators maintained contact with the employee (but did not elaborate on the specific nature of this communication) [15]. In one study, RTW coordinators met with the employee and their supervisor separately and together to brainstorm solutions for RTW and evaluate the eventual RTW plan [17]. Another article noted that RTW coordinators were responsible for providing advice and support to employees and workers’ families and for facilitating contact with the workplace and facilitated meetings with employers [16].

Actions and strategies—RTW Coordinator strategies and actions were expected to be addressed by qualitative studies. However, our final sample yielded no qualitative studies that had an analytic focus on RTW coordinators and mental health conditions and so we have no related findings. However, one study did consider the type of contact individuals had with a RTW coordinator [12].

Interventions—Three studies provided a multifaceted intervention that also included a RTW coordinator [13, 14, 16]. One study involved telephone interviews to compare features (including types of RTW Coordinator contact) that impacted self-efficacy for workers with physical and mental injuries [15]. One study involved the addition of a RTW coordinator to facilitate the RTW process between employees and employers [17].

Intervention Study Characteristics—Three articles describe outcomes observed for at least 1 year from the start of intervention [13, 14, 16]. One article described the observation as occurring at a 3-month follow-up [17]. Another study was cross-sectional and did not follow-up with participants [15]. Three studies focused on time to RTW [13, 14, 16]. Two used payroll data [13, 16] and one study considered the number of days spent on sickness absence, measured by considering a national registry noting sickness absence compensation and social transfer payments [14]. One study considered the feasibility of the intervention [17]. One study considered self-efficacy of workers with mental ill health [15].

Outcomes of interest—One study found that low stress (categorized as: not at all stressful, not very stressful or, a bit stressful), as opposed to high stress (categorized as quite a bit stressful or extremely stressful), contact with a RTW coordinator was associated with increased RTW self-efficacy for individuals with mental ill-health [15]. Two studies found that interventions including RTW coordinators for managing mental ill-health resulted in longer durations of time to RTW than conventional care [17]. One study found that for individuals with mental ill-health, there was a lower RTW rate in the intervention group (that used RTW coordinators) compared to conventional care [13]. However, another study found no difference in RTW rate for individuals with mental ill-health between control and intervention groups (with and without RTW coordinators) [16]. Further, a study found decreased RTW among individuals with mental ill-health in the control group (with no RTW coordinators) at the end of 2-years but increased cumulative sickness absence among those in the intervention group (with RTW coordinators) [14].

## Discussion

While both qualitative and quantitative articles were eligible for inclusion in this review, the only articles that were relevant to our study were quantitative. Additionally, although we included articles published between 2000 and 2018, the earliest article that was relevant for this review was published in 2009 [16]. Overall, the literature describing the role, strategies, actions and/or impact of RTW coordinators for return to work among individuals with mental ill-health is recent and limited (only 5 articles met inclusion criteria) and heterogeneous in study design, study purpose and findings.

### The Strategies and Actions of RTW Coordinators Managing Individuals with Mental Ill-Health

The articles in this review lacked a detailed description of the strategies and/or actions employed by RTW coordinators. No information was provided about specific strategies or actions utilized by RTW coordinators to facilitate RTW for individuals with mental ill-health. This paucity of information is consistent with findings from previous literature identifying that the strategies or actions undertaken by RTW coordinators was not well described [18]. One reason for this may be that requirements of this position are variable and may be evolving [5]. Additionally, the quantitative research design of studies meeting our inclusion criteria suggest that this topic area is lacking qualitative investigation, where these questions would more likely be assessed.

While the specific strategies and actions taken by RTW coordinators could not be determined, it is important to note that the background of coordinators may vary. It is possible that the background of RTW coordinators within this review do not capture the variation of RTW coordinators in this field. Overall, variation in RTW coordinator background and competencies may impact their scope of practice and range of understanding mental ill-health [5, 19].

### Sustained RTW and RTW Rate

Only one article included in this review considered sustained RTW, finding there was no difference in recurrent sickness absence between groups 2 years after a RTW intervention with a RTW coordinator [14]. Sustained RTW is an important outcome, due to the episodic and often chronic nature of mental ill-health among individuals who leave work and may be requesting leaves repeatedly. Thus, interventions that improve RTW sustainability for mental ill-health might be identified as valuable irrespective of the initial time to RTW. Further, among the studies included in this review, the presence of a RTW coordinator did not increase the RTW rate of individuals with mental ill-health; specifically, the RTW rate was lower in one study [13] and unchanged in another [16]. However, one study included in this review found a decreased RTW rate in the control group (with no RTW coordinators) at the end of 2 years [14]. Thus, the impact of a RTW coordinator on RTW rate was inconsistent across studies in this review. These findings, while not supporting the expectation that RTW coordinators will increase RTW rates, are in line with those of a systematic review of RTW coordination programs (not restricted to studies with RTW coordinators) that found no benefit of RTW coordination programs on RTW rate [20]. Additionally, as null results often are not published; we are not able to fully assess how many studies may have found that interventions with RTW coordinators are not more advantageous for rate of RTW than conventional approaches. However, while the RTW rate may not be reduced by interventions using RTW coordinators, previous research indicates that RTW coordination in general (e.g. multi-disciplinary interventions) decreases long term work disability and the cost of work absence, suggesting that the specific strategies and actions employed by RTW coordinators and the impact of these endeavours require more investigation [21].

### Time to RTW

Time to RTW was a key outcome of interest in this review. Of the five articles, three focused on duration of sickness absence as a primary outcome [13, 14, 16]. While minimal time to RTW may reduce absence costs, it is important to note that this is only one measure of the effectiveness of a RTW intervention and may be an insensitive measure to evaluate the quality of RTW interventions [16]. For example, a faster RTW may not improve quality of life or work functioning [22]. With respect to mental-ill health, it is possible that an increased time to RTW during an intervention might provide more sustainable supports to participants.

While studies were conducted with the expectation that the use of RTW coordinators decreases time to RTW, the current literature is inconclusive about the impact of RTW coordinators on reducing time to RTW for individuals with mental ill-health. When considering individuals on sick leave, one review found moderate evidence that disability duration is reduced when a RTW coordinator is a part of the intervention [21] while another review found no benefit for time to RTW [20]. Two articles included in this review found increased time to RTW for the intervention group that used RTW coordinators’ services [13, 17]. Possible explanations for these results are varied. First, precarious employment is increasing and, while it is unknown how many individuals with mental ill-health fall into this type of work, those with precarious work take longer to RTW [23]. Additionally, time to RTW is increased among individuals who take medication for mental ill-health [24]. Further, the specific health condition resulting in the work absence may be a relevant factor when considering time to RTW as some conditions (e.g. depression) are known to have longer RTW trajectories compared to milder forms of mental ill-health (e.g. distress) [25–27].

Therefore, while the overall time to RTW for interventions included in this review was elevated, this could be a reflection of the population of individuals with mental ill-health included in the studies. All of the articles included in this review combined individuals with various mental ill-health concerns. While the conditions included were all common mental health concerns, the needs and challenges will differ depending on the individuals and the conditions. Specifically, heterogeneity of mental ill-health was identified as a potential reason for increased time to RTW in the intervention group of one included article [13].

### Relevant Outcome Measures for Individuals with Mental Ill-Health

The outcomes currently being used to assess RTW coordinator interventions for populations with mental ill-health may not be appropriate. For example, one study considered the quality of interaction between the RTW coordinator and the worker: that an individual’s self-efficacy may be impacted by high stress or low stress interactions with a RTW coordinator [15]. Therefore, the impact of a RTW coordinator may vary depending on what strategies or actions are completed. Interestingly, none of the articles included in this review considered if the mental health of individuals was improved at the end of interventions. Overall, our review highlights the need to consider the specific mental health challenges of individuals involved in interventions in order to fully assess the value of RTW coordinators, and to further identify strategies that could improve the value of RTW coordinators when engaging with these individuals. For instance, strategies might include RTW coordinators working with mental health professionals and those with lived experience regarding what outcomes of interest for RTW may be of most value or relevance for sustainable RTW.

Overall, work is considered good for individual health and well-being [28]. However, literature supporting early RTW has placed an emphasis on decreasing time to RTW rather than improving quality of life [29, 30]. For individuals with mental ill-health, RTW coordinators may suggest that individuals seek psychological, psychiatric or pharmaceutical support, which may delay time to RTW [24]. Indeed, an inverse relationship may exist between improved mental health and early RTW.

Adding complexity to the position of RTW coordinators within this population is the often invisible and unpredictable nature of common mental illness, which may delay RTW [26, 27]. Also, it is possible that workplaces themselves may be a source of mental ill-health [31]. Research in both Canada and Australia has identified that psychological distress is pervasive within the majority of workplaces today [32, 33]. Further, poor psychological working conditions (such as work stress and low social support) can increase the risk of developing common mental health concerns [34, 35].

It is unclear that an early RTW is health-promoting or cost-effective when considering individuals who have left the workplace due to mental ill-health. Research has shown that cognitive behavioural therapy programs focused on work relevant solutions for mental health can reduce lost time and costs associated with work absence [30]. However, the optimal timing of RTW is unclear and RTW coordinators aiming for a quick work return may not be as effective when working with mental ill-health populations compared to those working with physical injury cases.

## Strengths and Limitations

To the best of our knowledge, this is the first review to explore the strategies, action and/or impact of RTW coordinators in relation to the return to work of individuals who have mental ill-health. Based on the limited literature identified, this review has clearly identified an important gap in the research that should be further considered.

A limitation of this work is the exclusion of articles considering serious mental health disorders. However, common mental disorders are the major source of disease burden from mental disorders. Furthermore, key differences between severe and common mental health conditions likely create disparate RTW trajectories [12, 36].

## Recommendations

While further work must be completed to fully understand the impact of RTW coordinators for mental ill-health populations, researchers should consider measuring populations with greater attention to within-sample heterogeneity and not grouping all common mental ill-health concerns together [25]. Additionally, qualitative research that focuses on the quality of RTW interventions should be completed. Overall, future research should evaluate the value of RTW coordinators by considering multiple outcomes that take into account not only the time and rate of RTW but also the quality of life for individuals receiving the intervention.

Of the four unique studies identified in our review there were two different names used to describe the position of RTW coordinators: Social Workers [13, 14, 16] and RTW Coordinators [15, 17]. While it has been suggested that consistent terminology should be used to ensure that the position fulfilled by a RTW coordinators is not misunderstood, the competencies that RTW coordinators have may still vary regardless of their title [34]. Some work has been done to identify the competencies important for RTW coordinators; however, this work was not specific to mental ill-health where additional competencies may be necessary [37]. Additionally, future research should give more detailed information about the competencies (training, background) as well as the strategies and actions used by RTW coordinators who engage with mental ill-health populations. This information will not only make it possible to establish best practices that can be employed for RTW but will also make it possible for researchers to compare coordinator positions between studies (to ensure that similar positions and approaches to the position are being compared).

Finally, due to the entirely quantitative design of studies included in this review, we recommend that future work include qualitative research approaches, which may be able to address questions about RTW Coordinators actions and strategies for managing the return to work of individuals with mental ill-health.

## Conclusion

There is a limited evidence base on the role of RTW coordinators when dealing with individuals who experience mental ill-health, and no evidence on the strategies and actions that they employ. Further, due to our small final sample size, the impact of RTW coordinators working with this population is still unknown. The sparse evidence available suggests that interventions for individuals with mental ill-health that employ RTW coordinators may be more time consuming than conventional RTW approaches and may not make a meaningful difference to the RTW rate. It is possible that RTW coordinators may improve quality of life for workers, but this outcome has not been addressed. More work should be done to assess the long-term outcomes of RTW coordination for mental ill-health as well as the experience of RTW coordinator support of these workers. Overall, research to date is insufficient to fully elucidate the strategies, actions and impact of RTW coordinators who support the RTW of individuals with mental ill-health. The value RTW coordinators convey to employers and workers when dealing with common mental health cases remains unclear.

## References

[1] Smetanin P, Stiff D, Briante C, Adair CE, Ahmad S, Khan M. Life at risk analysis of the impact of mental illness in Canada. RiskAnalytica, on behalf of the Mental Health Commission of Canada. 2011. https://www.mentalhealthcommission.ca/sites/default/files/MHCC_Report_Base_Case_FINAL_ENG_0_0.pdf.

[2] Gabriel P, Liimatainen M-R. Mental Health in the workplace Introduction executive summaries. International Labour Office Geneva. 2000. p. 1–27.

[3] Comission del Sante Mentale du Canada. Making the case for investing in mental health in Canada. Mental Health Commison of Canada. 2010. https://www.mentalhealthcommission.ca/English/system/files/private/document/Investing_in_Mental_Health_FINAL_Version_ENG.pdf.

[4] Birnbaum HG, Kessler RC, Kelley D, Ben-Hamadi R, Joish VN, Greenberg PE (2010). Employer burden of mild, moderate, and severe major depressive disorder: mental health services utilization and costs, and work performance. Depress Anxiety..

[5] Shaw W, Hong QN, Pransky G, Loisel P (2008). A literature review describing the role of return-to-work coordinators in trial programs and interventions designed to prevent workplace disability. J Occup Rehabil..

[6] Bohatko-Naismith J, James C, Guest M, Rivett DA (2015). The role of the australian workplace return to work coordinator: essential qualities and attributes. J Occup Rehabil..

[7] Carlsson L, Lytsy P, Anderzén I, Hallqvist J, Wallman T, Gustavsson C (2019). Motivation for return to work and actual return to work among people on long-term sick leave due to pain syndrome or mental health conditions. Disabil Rehabil..

[8] Ståhl C, Svensson T, Petersson G, Ekberg K (2009). The work ability divide: holistic and reductionistic approaches in Swedish interdisciplinary rehabilitation teams. J Occup Rehabil..

[9] Corbière M, Chézol MM, Bastien MF, Wathieu E, Bouchard R, Panaccio A (2019). Stakeholders’ role and actions in the return - to - work process of workers on sick—leave due to common mental disorders: a scoping review. J Occup Rehabil..

[10] MacEachen E, McDonald E, Neiterman E, McKnight E, Malachowski C, Crouch M, et al. Systematic review of the impact of return-to-work coordinators. Waterloo; 2019. 10.1007/s10926-021-09975-6 33881671

[11] Mechanic D, Bilder S, McAlpine DD (2002). Employing persons with serious mental illness. Health Aff..

[12] Ruggeri M, Leese M, Bisoffi G, Tansella M (2000). Definition and prevalence of severe and persistent mental illness. Br J Psychiatry..

[13] Martin MHT, Nielsen MBD, Madsen IEH, Petersen SMA, Lange T, Rugulies R (2013). Effectiveness of a coordinated and tailored return-to-work intervention for sickness absence beneficiaries with mental health problems. J Occup Rehabil..

[14] Martin MHT, Nielsen MBD, Pedersen J, Rugulies R (2015). Stability of return to work after a coordinated and tailored intervention for sickness absence compensation beneficiaries with mental health problems: results of a two-year follow-up study. Disabil Rehabil..

[15] Black O, Sim MR, Collie A, Smith P (2017). Early-claim modifiable factors associated with return-to-work self-efficacy among workers injured at work. J Occup Environ Med..

[16] Lander F, Friche C, Tornemand H, Andersen JH, Kirkeskov L (2009). Can we enhance the ability to return to work among workers with stress-related disorders?. BMC Public Health..

[17] van Oostrom SH, van Mechelen W, Terluin B, de Vet HCW, Anema JR (2009). A participatory workplace intervention for employees with distress and lost time: a feasibility evaluation within a randomized controlled trial. J Occup Rehabil..

[18] Gardner BT, Pransky G, Shaw WS, Hong QN, Loisel P (2010). Researcher perspectives on competencies of return-to-work coordinators. Disabil Rehabil..

[19] Franche RL, Baril R, Shaw W, Nicholas M, Loisel P (2005). Workplace-based return-to-work interventions: optimizing the role of stakeholders in implementation and research. J Occup Rehabil..

[20] Vogel N, Schandelmaier S, Zumbrunn T, Ebrahim S, de Boer W, Busse J (2017). Return-to-work coordination programmes for improving return to work in workers on sick leave. Chochrane Database Syst Rev..

[21] Franche RL, Cullen K, Clarke J, Irvin E, Sinclair S, Frank J (2005). Workplace-based return-to-work interventions: a systematic review of the quantitative literature. J Occup Rehabil..

[22] Pomaki G, Franche R-L, Murray E, Khushrushahi N, Lampinen TM (2012). Workplace-based work disability prevention interventions for workers with common mental health conditions: a review of the literature. J Occup Rehabil..

[23] Quinlan M, Mayhew C, Bohle P (2005). The global expansion of precarious employment, work disorganization, and consequences for occupational health: a review of recent research. Int J Heal Serv..

[24] Prang KH, Bohensky M, Smith P, Collie A (2016). Return to work outcomes for workers with mental health conditions: a retrospective cohort study. Injury.

[25] Nielsen MBD, Madsen IEH, Bültmann U, Christensen U, Diderichsen F, Rugulies R (2011). Predictors of return to work in employees sick-listed with mental health problems: findings from a longitudinal study. Eur J Public Health..

[26] Nielsen MBD, Bültmann U, Madsen IEH, Martin M, Christensen U, Diderichsen F (2012). Health, work, and personal-related predictors of time to return to work among employees with mental health problems. Disabil Rehabil..

[27] Roelen CAM, Norder G, Koopmans PC, Van Rhenen W, Van Der Klink JJL, Bültmann U (2012). Employees sick-listed with mental disorders: who returns to work and when?. J Occup Rehabil..

[28] Waddell G, Burton AK (2006). Is work good for your health and well-being?.

[29] Ellen M, Sue F, Kosny A, Chambers L (2016). A deliberation on ‘hurt versus harm’ logic in early-return-to-work policy. Policy Pract Heal Saf..

[30] Cullen KL, Irvin E, Collie A, Clay F, Gensby U, Jennings PA (2018). Effectiveness of workplace interventions in return-to-work for musculoskeletal, pain-related and mental health conditions: an update of the evidence and messages for practitioners. J Occup Rehabil..

[31] Åhlin JK, Lamontagne AD, Magnusson Hanson LL (2019). Are there bidirectional relationships between psychosocial work characteristics and depressive symptoms? A fixed effects analysis of Swedish national panel survey data. Occup Environ Med..

[32] Shain M (2012). Psychological safety at work: emergence of a corporate and social agenda in Canada. Int J Ment Health Promot..

[33] Hilton MF, Whiteford HA, Sheridan JS, Cleary CM, Chant DC, Wang PS (2008). The prevalence of psychological distress in employees and associated occupational risk factors. J Occup Environ Med..

[34] Memish K, Martin A, Bartlett L, Dawkins S, Sanderson K (2017). Workplace mental health: an international review of guidelines. Prev Med..

[35] Michie S, Williams S (2003). Reducing work related psychological ill health and sickness absence: a systematic literature review. Occup Environ Med..

[36] Jorm AF, Patten SB, Brugha TS, Mojtabai R (2017). Has increased provision of treatment reduced the prevalence of common mental disorders? Review of the evidence from four countries. World Psychiatry..

[37] Pransky G, Shaw WS, Loisel P, Hong QN, Désorcy B (2010). Development and validation of competencies for return to work coordinators. J Occup Rehabil..

